# Biosorption of Congo Red from aqueous solution by crab shell residue: a comprehensive study

**DOI:** 10.1186/s40064-016-2113-9

**Published:** 2016-04-27

**Authors:** Tamtam Mohan Rao, Vudata Venkata Basava Rao

**Affiliations:** Department of Chemical Engineering, Bapatla Engineering College, Bapatla, India; Faculty of Technology, University College of Technology, OU, Hyderabad, India

**Keywords:** Adsorption, Congo Red, Crab shell, Isotherms, Kinetics, Thermodynamics, RSM

## Abstract

The abundantly available bio waste, crab shell powder was used as an adsorbent for the removal of pollutants like Congo Red. The morphological, textural and chemical characterization of the biomass was done with SEM, XRD, EDS and FT-IR studies. The nature and mechanism of the process were determined from equilibrium, kinetic and thermodynamic studies. The results exhibited that the bio waste surface is fractured, rough and porous. It is composed of various surface functional groups which attracts organic pollutants. Equilibrium studies conclude Adsorption is a favorable process and it is a monolayer covering the surface. The maximum adsorption capacity, given by non-linear Langmuir isotherm was 124.9 mg/g. In kinetic studies pseudo-second order model best described the sorption kinetics compared to other models. Thermodynamic studies conclude that the process is spontaneous, endothermic and a physical adsorption.

## Background

By 2030, the world’s population is expected to increase to 8.3 billion and total seafood demand is estimated to be 183 million tons, while the estimated global seafood market is predicted to reach US$ 100 billion per annum. In addition, the world demand for seafood is currently increasing by 3 % every year. High-end sea food products, such as lobster and crab, are consumed more often at restaurants than at home. Crab continues to compete well against other seafood proteins, ranking in the top 10 highest consumed sea food products, while being the most expensive species on the list. American per capita crab consumption amounts to 0.7 pounds per year. In 2012, US alone imported 5.4 million pounds in a month. Only 20–30 % of the weight of the crab is processed for human food consumption. The remaining 70–80 % is generally discarded, causing surface and ground water pollution and increase of BOD and COD (Gupta and Suhas 2009).

At present, total dye production is estimated as 7 × 10^5^–1 × 10^6^ tons per year. More than 1 × 10^5^ commercial dyes are produced, of which nearly 70 % are azo dyes. About 66 % of the total dye stuff produced is used in the textile industry, where nearly 100 L of water is required to process every kilogram of dye, and 10–15 % of the used dyes enter the environment through effluents. Most dyes are carcinogenic and cause skin and eye irritation. Presence of dyes in water bodies is highly objectionable on aesthetic grounds, and it disturbs the aquatic ecosystem by interfering with light transmission (Grag et al. 2003; Sirianuntapiboon and Srisornak 2007; Rangabhashiyam et al. 2014).

Congo Red (CR) is the first synthetic azo dye produced for dying cotton directly. It is used in a number of industrial activities, and consequently, found commonly in effluents (Vimonses et al. 2009; Purkait et al. 2007). Treatment of such effluents is difficult because CR is resistant to bio and photo-degradation due to its complex aromatic structure, physicochemical, thermal, and optical stability properties (Pielesz 1999; Smaranda et al. 2011).

Various physical, chemical, biological, acoustic, radiation, and electrical methods are adopted for dye removal. Of these, the biological method is commonly used because it is cost-competitive and suitable for a variety of dyes. However, it has the disadvantages of large space and longer process times requirements and less flexibility in design and operation (Zvezdelina and Nedyalka 2012; Robinson et al. 2011). Adsorption, generally using activated carbon, is considered the best alternative. However, because activated carbon is expensive and difficult to regenerate, it is important to find cheaper and environmentally friendly alternatives (Bhattacharyya and Sarma 2003). The concept of industrial ecology encourages the use of waste from one industry in the processes of another. In recent years, usage of biomass and solid waste as low-cost adsorbents has gained much attention (Ali and Gupta 2007).

In this study, we discuss the use of solid waste from the sea food industry for the treatment and elimination of toxics from waste water. Crab shell powder (CSP) has several advantages for use as an adsorbent, including ease of availability, low cost, and high biocompatibility. We extend our investigation to estimate the limitations and binding mechanism through kinetic, isothermal, and thermodynamic studies.

## Methods

### Materials

Congo Red, NaOH, and HCl supplied by Merck (Mumbai, India).

### Preparation of CSP

Crab shells were collected directly from a local market (Bapatla, Andhra Pradesh, India), washed with tap water to remove slime and other debris, rinsed with distilled water, and then dried in an oven at 60 °C to a constant weight. The dried shells were crushed to a particle size of 40–120 µm. Acid-base treatments were given using 1N HCl and 1N NaOH, followed by repeated washing with deionized water, and drying in an oven at 60 °C over night. The treated particles were crushed again, separated using British Standard Sieves (BSS), and stored in dry vacuum packs to prevent moisture penetration for ready use as an adsorbent.

### Instruments

- Digital weighting balance—SHIMADZU–AX200
- Digital pH meter–ELICO-L 1 612
- Temperature controlled rotating orbital shaker— REMI CIS 24 BL
- High speed centrifuge-REMI C 24
- UV—visible spectrophotometer—SYSTRONICS–117

### Characterization

The surface morphology of CSP was examined by scanning electron microscopy (SEM, Carl Zeiss, EVO-18) equipped with EDS analyser. IR spectra of CSP obtained with a SHIMADZU, FTIR 8400SFourier transform infrared spectrometer FTIR.

### Calculations

The amount of dye adsorbed at equilibrium *q*_*e*_ (mg/g) was calculated using the following equation

1 $$ q_{e} = \frac{{\left( {C_{0} - C_{e} } \right)V}}{w} $$

where *C*_0_ and *C*_*e*_ (mg/L) are concentrations of dye solution at initial and equilibrium, respectively, V (L) the volume of the solution, and *w*(g) is the mass of the adsorbent used. A similar procedure can be adopted for kinetic studies where samples are withdrawn at different time intervals.

The amount of dye adsorbed at time t, *q*_*t*_ (mg/g), was calculated using

2 $$ q_{t} = \frac{{\left( {C_{o} - C_{t} } \right)V}}{w} $$

The extent of adsorption is expressed in percentage as removal efficiency (%R) and is calculated using

3 $$ \% {\text{R}} = \frac{{\left( {C_{o} - C_{t} } \right)}}{{C_{o} }} \times 100 $$

## Results and discussions

### Parameters

#### Effect of particle size

**Fig. 1:**
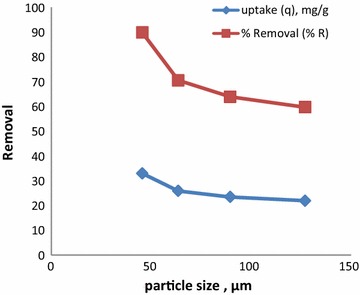
Effect of particle size on CR removal using CSP

The surface area available for adsorption is inversely proportion alto particle size of the sorbent and is an important controlling parameter in the adsorption process. The effect of particle size on CR adsorption was studied using samples of four different average particle sizes: 45.50, 63.50, 89.50, and 127.00 µm and the results are presented in Fig. 1. It was established that adsorption of CR decreased with increase in particle size of CSP. This is due to increase in the surface area with particle size reduction for a fixed amount of absorbent. These findings are consistent with related studies done on removal of Direct Red 12B using garlic peel (Asfaram et al. 2014), adsorption of CR on *E. Crassiperoots* (Wycliffe et al. 2014). This relationship indicates that powdered fine adsorbent would be advantageous over granular particles for adsorption of CR.

#### Effect of dosage

**Fig. 2:**
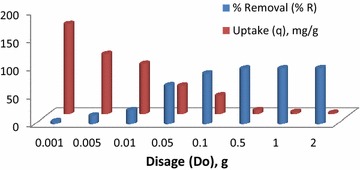
Effect of dosage on CR uptake (q) and % removal (% R) using CSP

The effect of adsorbent dosage is significant from an economic point of view (Mustafa et al. 2014; Salleh et al. 2011). It is an important process parameter that helps determine the adsorption capacity of an adsorbent for a given set of operating conditions. Normally, increasing dosage causes an increase in %R and a decrease in dye uptake. The effect of CSP dosage on the CR removal is presented in Fig. 2. Removal efficiency depends only on initial and final concentrations and doesn’t account for the mass of adsorbent used. An increase in dosage increases both, the population of active sites and the surface area. This enhances the rate of mass transfer and causes a subsequent increase in %R. For a fixed concentration of CR, sharing of sorbate molecules decreases with increase in active sites. Hence, dye uptake decreases with dosage.

#### Effect of initial dye concentration

**Fig. 3:**
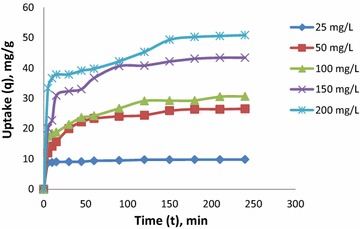
Effect of time and concentration on CR removal using CSP

An increase of uptake is observed with increase in initial dye concentration due to an enhanced driving force for mass transfer. Experiments were conducted at different initial concentrations (25, 50, 100, 150 and 200 mg/L), and the observed uptake at different time intervals is shown in Fig. 3. The rate of uptake is maximum in the beginning; decreases with time, and ultimately stabilizes after t = 150 min (all experiments were conducted for 240 min to ensure equilibrium). The concentration of both phases changes simultaneously with time; and hence, the concentration difference is the driving force for mass transfer. This is why uptake decreases with time and is constant at equilibrium.

#### Effect of pH

**Fig. 4:**
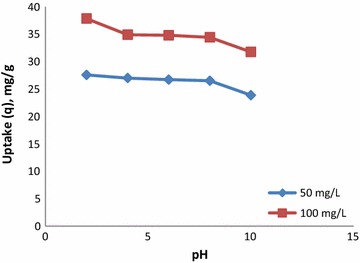
Effect of pH on CR removal using CSP

Effect of pH was studied by conducting experiments using a 100 mg/L solution and 2 g/L dosage at 30 °C, and the results are shown in Fig. 4. Low pH leads to an increase in H^+^ ion concentration in the system and the surface of the CSP acquires positive charge by absorbing H^+^ ions. As a result, significantly strong electrostatic attraction appears between the positively charged CSP surface and anionic dye molecules, leading to higher adsorption. High pH leads to an increase in the negatively charged sites and decrease in the positively charged sites. A negatively charged surface site on the CSP does not favor the adsorption of anionic dye molecules due to electrostatic repulsion. Furthermore, adsorption of the CR dye is lower in alkaline media owing to the competition from excess OH^-^ ions with the anionic dye molecule for the adsorption sites.

### Isothermal studies

Isothermal studies help determine the maximum adsorption capacity and the mechanism of surface coverage on the adsorbent. If the adsorbent and adsorbate are in contact for a long time, equilibrium is established between them and the equilibrium relation is called an isotherm. There are several models to predict the equilibrium distribution, and the Langmuir and Freundlich models are the most widely used to describe the adsorption isotherm. The non linear and linear forms of these are as follows:

4 $$ q_{e} = \frac{{q_{max} K_{F} C_{e} }}{{1 + K_{F} C_{e} }} $$

5 $$ \frac{{C_{e} }}{{q_{e} }} = \frac{{C_{e} }}{{q_{max} }} + \frac{1}{{K_{F} q_{max} }} $$

6 $$ q_{e} = K_{f} C_{e}^{1/n} $$

7 $$ \ln q_{e} = \ln K_{f} + 1/n\ln C_{e} $$

The linear forms of Langmuir and Freundlich isotherms (Eqs. 5, 7) were tested, and the calculated values of isotherm parameters are given in Table 1. The comparison of correlation coefficients (R^2^) of both equations indicates that the data is better represented by the Langmuir model. This suggests monolayer coverage of the surface of CSP by CR molecules. A dimensionless constant ‘R_L_’, called separation factor or equilibrium parameter, is calculated using the following equation in order to predict whether an adsorption system is favorable or unfavorable (Tien 1994):

8 $$ R_{L} = \frac{1}{{1 + K_{L} C_{o} }} $$

where K_L_ is the Langmuir constant (dm^3^/mol) and C_0_ is the highest initial dye concentration (mol/dm^3^). The values of R_L_ calculated from Eq. 8 are incorporated in Table 1, and indicate that adsorption is favorable (Safa et al. 2005).

**Table 1:** Isothermal parameters from linear models

Model	Langmuir			Fruendlich			R_L_
Parameter	R^2^	q_max_ (mg/g)	K_L_ (L/mg)	R^2^	n	K_F_ (mg/g) (L/mg)^1/n^	
Value	0.98773	119.4743	0.016711	0.971	1.6639	4.043	0.1–0.29

**Fig. 5:**
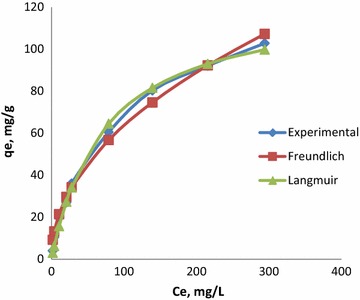
Comparison of non linear forms of isotherms for the sorption of CR using CSP

Non-linear regression was used to establish the best fit of experimental data for non linear models of Langmuir and Freundlich expressions, as presented by Eqs. 4 and 6. A trial and error procedure was applied to determine the parameters of these models. A comparison of the results of non-linear models is presented in Fig. 5 and the parameters obtained are presented in Table 2. The value of q_max_ obtained from non linear Langmuir isotherm is 124.9 mg/g, which is very close to that of experimentally determined value of 130 mg/g.

**Table 2:** Isothermal parameters from non-linear models

Model	Langmuir			Fruendlich			R_L_
Parameter	R^2^	q_max_ (mg/g)	K_L_ (L/mg)	R^2^	n	K_F_ (mg/g) (L/mg)^1/n^	
Value	0.999467	124.9027	0.013545	0.986	2.066	6.852	0.013–0.33

### Kinetics

Design of adsorption process depends on the rate which can be estimated from kinetic study. The kinetics plays a key role in choosing the best operating conditions for the full-scale batch process. It also gives an idea about mechanism of mass transfer and rate controlling steps.

Batch sorption kinetics for the removal of CR by CSP has been studied in terms of pseudo-first-order and second order kinetics models, and intra-particle and film diffusion models. The pseudo-first-order rate expression of Lagergren based on solid capacity is generally expressed as (Lagergren 1898):

9 $$ \ln \left( {q_{e} - q_{t} } \right) = lnq_{e} - K_{1} t $$

where q_e_ and q_t_ are the amounts of solute adsorbed a equilibrium and at time t (min) respectively. K_1_ is the rate constant (min^−1^). A straight line for the plot of ln(q_e_ − q_t_) versus t would suggest the applicability of this kinetic model for the experimental data. The pseudo-first-order rate constant K_1_ and equilibrium adsorption density (q_e_) were calculated from the slope and intercept of this line and are given in Table 3.

**Table 3:** Kinetic parameters from linear models

Conc. (mg/L)	I-Order			II-Order			q_e_,_exp_ (mg/g)
	R^2^	K_1_ (min^−1^)	q_e_ (mg/g)	R^2^	K_2_ (g/min)	q_e_ (mg/g)	
25	0.979	0.02	1.393753	0.999	437.6322	9.90099	10.54
50	0.874	0.019	12.01313	0.997	2581.131	27.02703	26.65278
100	0.911	0.01	20.78019	0.992	2496.879	33.33333	33.65154
150	0.972	0.009	16.97939	0.996	10,989.98	45.45455	45.7284
200	0.906	0.018	39.17348	0.992	10,119.41	55.55556	60.52469

**Fig. 6:**
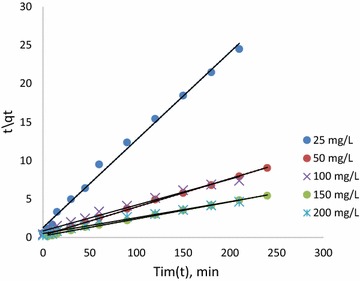
Pseudo second order kinetics for CR removal using CSP

A pseudo-second-order kinetic model of Ho and McKay (1999) is given as the following:

10 $$ \frac{t}{{q_{t} }} = \frac{1}{{K_{2} q_{2e}^{2} }} + \frac{t}{{q_{2e} }} $$

where, K_2_ is the rate constant for pseudo-second-order reaction (g/min). A plot of t/q_t_ versus t should give a linear relationship for its applicability. The rate constant (K_2_) and equilibrium uptake (q_e_) were obtained from the intercept and slope of linear plots (Fig. 6) generated with the experimental data, were given in Table 3.

The q_e_ values estimated from the pseudo first-order kinetic model significantly differed with that of experimental values. Where as they agreed well with the estimates of pseudo second-order kinetic model with high correlation coefficient (R^2^ ≥ 0.994). Hence, the kinetics for sorption of CR on to CSP is analogous to pseudo second-order type. Similar phenomena were also observed in the adsorption of Congo red on activated carbon, calcium-rich fly ash and CaCl_2_ modified bentonite (Purkait et al. 2007; Lian et al. 2009).

In general mass transport through solid/liquid interface takes place by three different mechanisms, film diffusion, intra particle diffusion, and mass action. Mass action is a very rapid process and can be negligible for physical adsorption. Thus, the kinetic process of adsorption is controlled by either liquid film diffusion or intra particle diffusion or both.

The structure of the adsorbent and its interaction with the diffusing adsorbate (Intra particle diffusion) influence the rate of transport, where the solute movement is a function of concentration gradient (Wu et al. 2009), and the rate constant (k_i_) can be determined by Intra-particle diffusion model.

11 $$ {\text{q}}_{\text{t}} = {\text{k}}_{\text{i}} \surd {\text{t}} + {\text{C}} $$

Weber Morris found that in many adsorption cases solute uptake varies almost proportionally with t^1/2^ rather than with the contact time t. Where q_t_, C, k_i_ refers to the amount of dye adsorbed in mg/g at time t, parameter indicating the boundary layer effect and Intra-particle diffusion rate constant (mg/g. min^1/2^). A plot between the amount of dye adsorbed and square root of time gives the rate constant. The plot should be the straight line passing through the origin if the intra particle diffusion is the sole rate limiting step (Kumar et al. 2007).

**Fig. 7:**
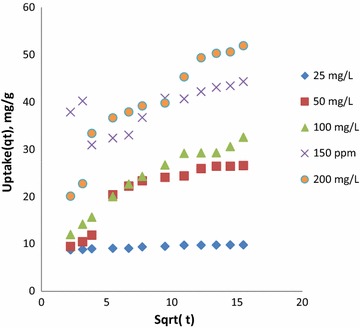
Intra particle diffusion kinetics for CR removal using CSP

Figure 7 represents intra particle diffusion plots generated from the experimental data. The points at all conditions were scattered and don’t represent a straight line. Even if a straight line is drawn using least square method, it doesn’t pass through origin. This indicates intra particle diffusion is not only the limiting step. But it is more prominent at higher concentrations and also tending to multi step due to increased concentration difference.

The film diffusion mass transfer rate equation is presented as

12 $$ \ln \left( {1 - {\text{q}}_{\text{t}} /{\text{q}}_{\text{e}} } \right) = - R^{\prime }{\text{t}} $$

$$ R^{{\prime }} = 3D_{e}^{{\prime }} /\left( {\Delta {\text{r}}_{0} {\text{r}}_{0} k^{{\prime }} } \right) $$

where *R′* (1/min) is the liquid film diffusion constant, *D′*_*e*_ (cm^2^/min) is the effective liquid film diffusion coefficient, r_0_ (cm) is the radius of the adsorbent beads, Δr_0_ (cm) is the thickness of the liquid film, and *k′* is the equilibrium constant of adsorption.

A plot of −ln(1 − q_t_/q_e_) vs. t should be a straight line with a slope *R′* if the liquid film diffusion is the rate limiting step. The liquid film mass transfer equation has been successfully applied to model several liquid adsorption systems.

**Fig. 8:**
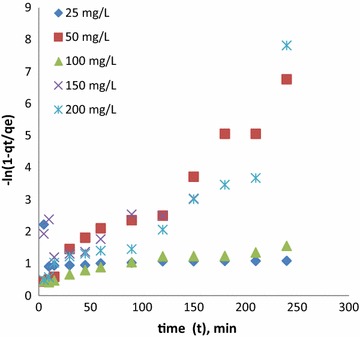
Film diffusion kinetics for CR removal using CSP

Experimental data was tested for film diffusion kinetics, and represented as Fig. 8. Results indicate linear trend in the beginning period of time and deviation lateron, confirm liquid diffusion is not only the limiting step and there is a chance of mixed kinetics.

### Thermodynamic studies

The thermodynamic parameters such as the Gibbs free energy (ΔG^0^), the enthalpy (ΔH^0^) and the entropy (ΔS^0^) can be determined by using the following equations:

13 $$ K_{c} = \frac{{C_{A} }}{{C_{s} }} $$

14 $$ \Delta G^{o} = - RT\ln K_{c} $$

15 $$ \ln K_{c} = - \frac{{\Delta G^{o} }}{RT} = \frac{{\Delta H^{o} }}{RT} + \frac{{\Delta S^{o} }}{R} $$

where K_c_ is the equilibrium constant, C_A_ and C_S_ are equilibrium concentrations (mg/g) of the dye in solid and liquid phases. T is the temperature in Kelvin and R is the gas constant. The linear plots of *lnK*_*C*_vs1/*T* yields ΔH^0^ and ΔS^0^ in the form of slope and intercept, respectively. Energy and entropy concentrations accounts for spontaneous nature of the process (Li et al. 2010). The plots were generated for the data of experiments conducted at different temperatures and the calculated values of thermodynamic parameters for the adsorption of CR onto CSP were given in Table 4.

**Table 4:** Thermodynamic parameters

Conc. (mg/L)	ΔH° (kJ/mol)	ΔS° (kJ/mol)	ΔG° (kJ/mol)			
			20 °C	30 °C	40 °C	50 °C
25	20.22796	0.143417	−21.6274	−23.625	−24.5738	−26.0701
75	24.51799	0.148571	−19.0691	−20.4903	−22.0466	−23.5067
100	19.21365	0.132109	−19.1127	−21.5289	−22.1606	−23.2494
150	24.74246	0.144497	−20.2918	−19.1767	−20.2862	−22.0822
200	12.34629	0.108165	−19.6155	−20.3043	−21.0846	−23.0165

The adsorption is endothermic since the values of ΔH^0^ are positive. The values of enthalpies at all concentrations are less than 25 kJ/mol indicates the interfacial interaction is by physical forces and hence, it is physical adsorption. Positive values of ΔS^0^ indicate increased randomness at solid liquid interface thereby increase in adsorbate concentration on solid phase during sorption process. All the values of ΔG^0^ are less than −27 kJ/mol. The negative values indicate feasibility and spontaneity of the process.

### Characterization

**Fig. 9:**
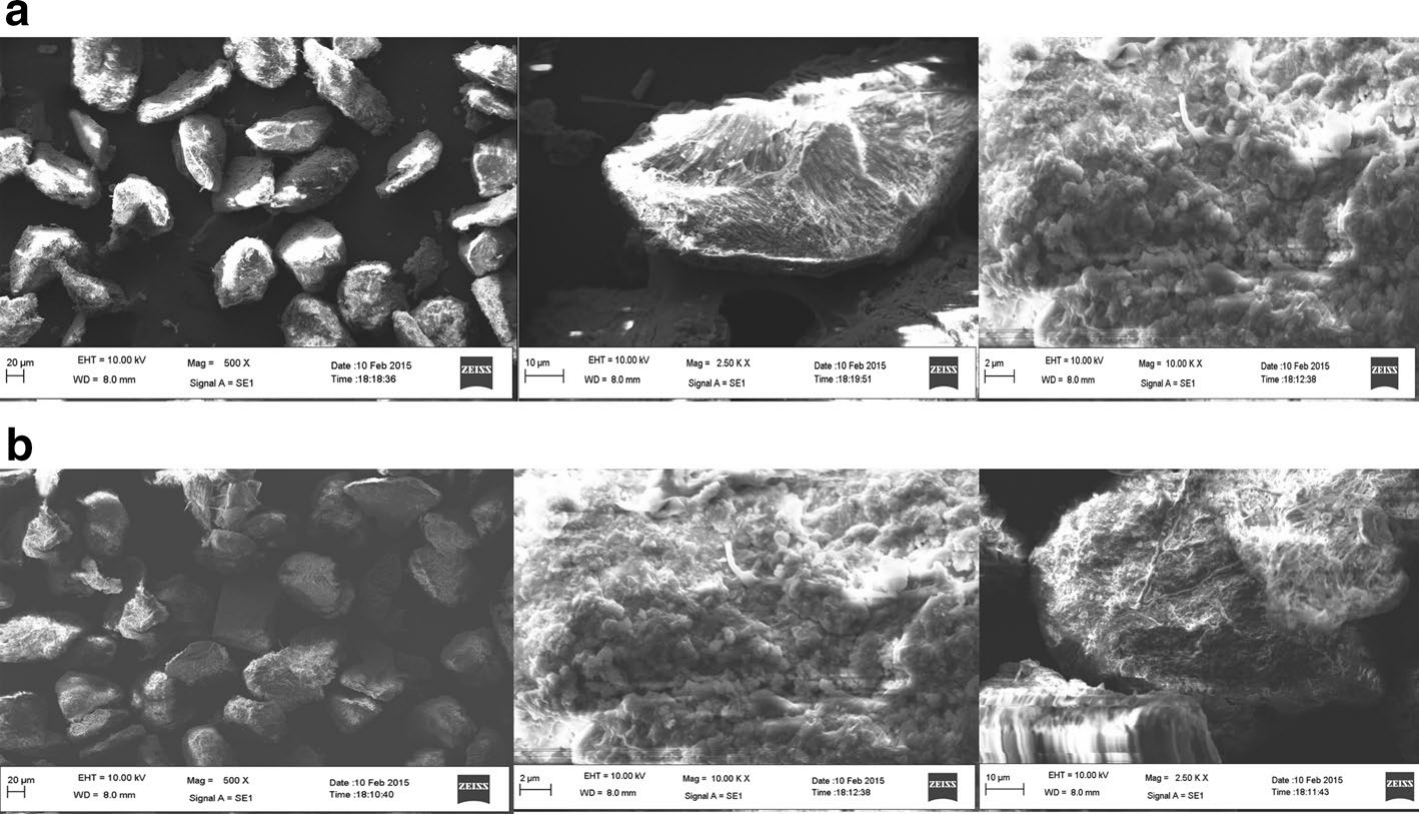
**a** Scanning electron microscope images of CSP adsorption at a magnification of ×600, ×2.5k and ×10k. **b** Scanning electron microscope images of CSP after adsorption at a magnification of ×600, ×2.5k and ×10k

Morphology of CSP is shown in the Fig. 9a. The forms and shapes of the particles are very irregular and its surface is fractured, rough and porous. The distribution of the particle sizes is not uniform. SEM image after adsorption is shown in Fig. 9b. The change in morphology is due to accumulation of CR on CSP surface.

**Fig. 10:**
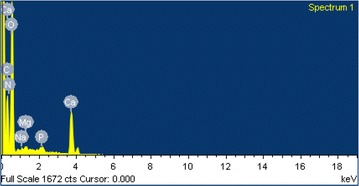
EDS spectra of CSP

Crab shell is primarily composed of CaCO_3_, Ca_3_(PO_4_)_2_, and chitin (Laughlin et al. 1973). The elemental analysis was obtained from EDS spectra as shown in Fig. 10 and the values were given in Table 5. Apart from O (54.17 %), the major elements are Ca, C and N with 16.86 , 15.62 and 12.72 %. The major minerals identified from EDS spectrum were calcium, phosphorus and magnesium (Fig. 10).

**Table 5:** EDS for elemental analysis of CSP

Element	wt%	at.%
C	15.62	21.53
N	12.72	15.03
O	54.17	56.06
Na	0.16	0.11
Mg	0.35	0.24
P	0.12	0.07
Ca	16.86	6.96

**Fig. 11:**
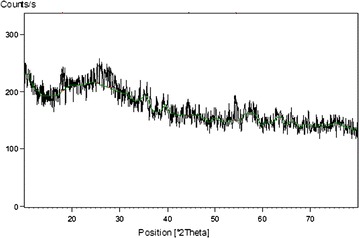
XRD spectra of CSP

XRD: X-ray diffraction of CSP was given in Fig. 11. It shows amorphous nature of the powder. Calcium is by far the most common mineral and much of it is present as calcium carbonate and in an amorphous form. Amorphous calcification may be related to the relative proportion of P to Ca -with only a few percent of phosphate ions in the exoskeleton inhibiting the crystallization of calcium carbonate FTIR.

**Fig. 12:**
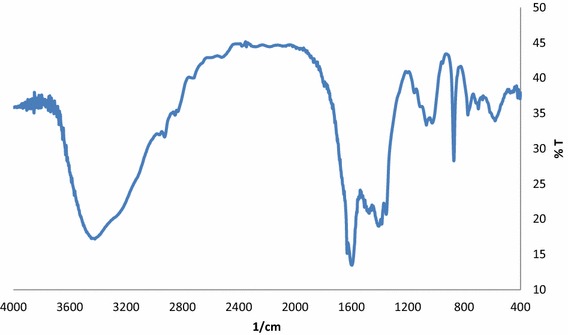
FTIR spectra of CSP

Various absorption bands within the 4000–400 cm^−1^ range were recorded in the FTIR spectra of CSP (Fig. 12). The bands at 3421–3439 cm^−1^ could be assigned to N–H, O–H and NH_2_ associated with chitin and chitosan. The presence of n acetyl group was proven by peaks at 1597, 1629 cm^−1^ which corresponds to C=O vibration. Peaks in the range of 1350–1400 cm^−1^ indicate CH_3_ stretching in NHCOCH_3_. Peaks near 1400 cm^−1^ is related to calcium carbonate. Small broad peaks at 1068, 1026 cm^−1^ indicate the presence of phosphate groups in calcium phosphate. The absorption bands within the 1422–603 cm^−1^ region confirmed the presence of CH_3_, CH_2_ and CH groups as well as the primary and secondary OH groups, attached to the pyranose ring, and the oxygen atoms in ether groups.

## Conclusions

Sea food industry is one of the major sources of solid waste posing disposal problems. It can be used to remove organic pollutants emanating from various industries. In the present study, Crab shells were powdered and used to remove Congo red dye from water effluent. CSP is a rough and porous material primarily composed of calcium sulfate, calcium carbonate and chitin. It has functional groups of amine, hydroxyl and acyl groups along with sulfates and carbonates. CR diffuses and binds to CSP in aqueous phase, favored by intermolecular forces. The maximum adsorption capacity given by non-linear Langmuir isotherm was 124.9 mg/g. Both intra particle and film diffusion limits the rate while pseudo second order is the kinetic model representing the process. Thermodynamic studies reveal the removal was spontaneous and endothermic in nature.
